# PCMT1 as a prognostic marker in breast cancer

**DOI:** 10.1007/s12094-024-03695-y

**Published:** 2024-09-05

**Authors:** Patryk Rogaczewski, Michał Janiak, Jędrzej Borowczak, Łukasz Szylberg

**Affiliations:** 1 Department of Tumor Pathology and Pathomorphology, Oncology Centre in Bydgoszcz ul. Romanowska, Prof. Franciszek Łukaszczyk Memorial Hospital, 85-796 Bydgoszcz, Poland; 2 Department of Obstetrics, Gynaecology and Oncology, Collegium Medicum in Bydgoszcz, Nicolaus Copernicus University in Torun, Bydgoszcz, Poland

**Keywords:** PCMT1, Cancer, Triple-negative breast cancer, Prognosis, Survival

## Abstract

**Introduction:**

Triple-negative breast cancer (TNBC) is one of the most aggressive cancers in women, therefore it is necessary to determine novel prognostic markers to estimate survival and advancement the treatment of the disease. Recently, PCMT1, a protein mediating TNBC immune infiltration, has gained attention as a potential therapeutic target. The aim of the study was to demonstrate the relationship between PCMT1 protein overexpression as a prognostic indicator for patients with TNBC cancer and patient survival.

**Materials and methods:**

The study included 64 samples collected from 64 TNBC patients. We used the ImageJ software with the IHC Profiler driver for image analysis. To improve the reliability of the results, we expanded the analysis by including The Cancer Genome Atlas cohort.

**Results:**

We observed strong PCMT1 immunoreactivity in breast cancer samples and PCMT1 expression in TNBC was significantly higher than in the control group. Patients with high PCMT1 expression had a significantly lower overall survival rate (60.62% vs. 90.35%, respectively) than patients with low PCMT1 expression. In our study and TCGA groups, PCMT1 expression did not correlate with lymph node involvement and distant metastases but correlated with tumor stage. The results obtained in a larger TCGA group are consistent with those in our research group. Overexpression of PCMT1 was a prognostic marker of shorter survival in patients with TNBC.

**Conclusions:**

The overexpression of the PCMT1 protein in triple negative breast cancer significantly correlated with shorter overall survival. The confirmed association could be a potential prognostic biomarker for patients with TNBC.

## Introduction

Breast cancer accounts for 20–30% of all cancer cases in women and is the most often diagnosed malignancy in females [1]. Its subtype, called triple-negative breast cancer (TNBC), is characterized by the lack of human epidermal growth factor receptor 2 (HER2), estrogen receptors (ER) and progesterone receptors (PR), and accounts for a disproportionately high percentage of breast-cancer fatalities, while constituting only ~ 15% of all invasive cases [2]. TNBC continues to have a bad prognosis despite the fact that the treatment of patients with breast cancer has recently improved. TNBC is distinguished by significant intra-tumor heterogeneity, frequent acquisition of treatment resistance, and the lack of biomarkers, which make treatment difficult [3].

Protein L-isoaspartate (D-aspartate) O-methyltransferase (PCMT1) type II, sometimes referred to as PIMT, is a repair enzyme that recognize and transform L-isoaspartyl and D -aspartyl by starting the process of converting aspartyl residues that have spontaneously isomerized back to their original form [4]. The human PCMT1 protein is a monomeric enzyme composed of two isoforms that were combined during alternative splicing [5]. PCMT1 has been linked to a number of RNA processing activities, such as controlling mRNA nuclear export to promote protein translation [6]. Cellular aging and microenvironmental stress are linked to spontaneous protein deamidation and isomerization, which produces aberrant L-isoaspartyl (isoAsp) residues from normal L-aspartyl and L-asparaginyl residues [7, 8].

PCMT1 regulates tumor progression and potentially influences the activity of signaling pathways that may become therapeutic targets for the treatment of human cancers. However, the role of the PMCT1 protein is not yet fully explained [9–11]. The role of PCMT1 as a predictive marker was suggested not only in breast cancer but also in other malignancies [11–14]. High PCMT1 levels predict poor prognosis in surgically removed lung adenocarcinoma and its overexpression enhances the growth of glioblastoma, which translates into reduced patient survival. High expression of PCMT1 was present in bladder cancer cells and associated with a poorer prognosis for patients. These studies show that PCMT1 is of great importance in the development of a wide variety of malignancies [12–14].

Increased PCMT1 expression is associated with a bad prognosis in breast cancer patients; however, its prognostic value in TNBC is not fully known [11]. The studies conducted so far show that PCMT1 expression is correlated with the advanced tumor stage of breast cancer and that the association with immune-infiltration biomarkers suggest that it may be a prognostic marker of breast cancer. In addition, increased PCMT1 expression may be associated with reduced patient survival and poor prognosis [12].

This study aims to demonstrate the relationship between PCMT1 overexpression, TNBC pathologic malignancy features of the tumor and survival of patients. We will also analyze the role of PCMT1 as a prognostic indicator for patients with TNBC cancer and patient survival.

## Materials and methods

### Clinicopathological data

Tissue specimens were collected from 64 patients diagnosed with TNBC and treated in the Oncology Centre between 2013 and 2020. We used normal tissue from the tumor area as a control group for each of the same cases in the study group. This allowed for a comparative analysis between tumor and normal tissue. The collected clinical data included age, dates of treatment, stage T, recurrence, lymph-node metastasis, and patient survival status (Table 1). The study was conducted following the Declaration of Helsinki, and the protocol was approved by the Bioethics Committee (KB 476/2021).

**Table 1:** Clinicopathological characteristics of study group

Variables		N (%)
Age	Mean	58.2 years (range 32–85)
Mean	Female	64 (100%)
Clinical stage	IA	14 (21.88%)
	IIA	39 (60.94%)
	IIB	8 (12.5%)
	IIIB	3 (4.69%)
Stage	T1	15 (23. 44%)
	T2-T4	49 (76.56%)
Lymph-node metastases	N0	44 (68.75%)
	N1-N3	18 (28.13%)
	NX	2 (3.12%)
Neoadjuvant therapy	No	63 (98.44%)
	Yes	1 (1.56%)
Adjuvant therapy	No	1 (1.56%)
	Yes	63 (98.44%)
Recurrence	Yes	10 (15.62%)
	No	54 (84.38%)
Mean recurrence time		32.89 months
Disease course	Alive	50 (78.12%)
	Dead	14 (21.88%)
Median follow-up time		90 months

### Sample staining

The expression of PCMT1 was determined using immunohistochemistry (IHC) assays following the standard diagnostic protocol. In the beginning, standardization and optimization of the IHC method were performed on a recommended tissue based on the antibody datasheet and reference sources [15]. In brief, 3 μm thick sections of the tissue arrays were baked for 1 h at 60 °C before xylene deparaffinization and subsequent rehydration through graded ethanol (99.8, 96, 90 and 80%). Tissue sections were incubated with a primary rabbit monoclonal anti-PCMT1 antibody (ab97446). Primary antibodies were visualized using either the UltraView Universal DAB Detection Kit (Roche Diagnostics/Ventana), followed by color development using 3,3-diaminobenzidine. The slides were counterstained with Hematoxylin II for 12 min and Bluing Reagent for 4 min. Finally, tissue sections were dehydrated in increasing ethanol concentrations (80, 90, 96, and 99.8%), cleared in xylenes (I–IV), mounted using a mounting medium, and examined.

### Image acquisition and analysis

**Fig. 1:**
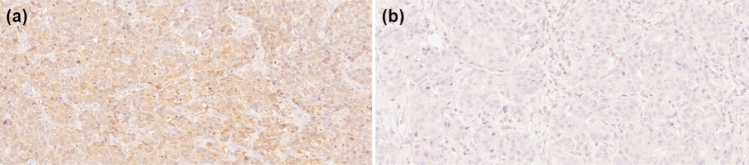
Representative cross-sectional staining patterns in TNBC study group; **a** high PCMT1 expression, **b** low PCMT1 expression

Images were captured using an optical microscope at × 20 magnification with a color video camera plugin to a computer. Researches selected the most representative regions for the further analysis. We used ImageJ 1.53j version (NIH, Bethesda, MD, USA) (Java 1.8.0_172) software with the IHC Profiler plugin to carry out image analysis. The background was subtracted and three measurements were made for each case in three independent, representative areas of the preparation in the DAB-Stain image. From the values obtained, the average was calculated. During the whole image analysis the clinical data were blinded (Fig. 1).

### In-silico analysis

The FPKM values of PCMT1 expression were obtained through The Human Protein Atlas (www.proteinatlas.org) and the TCGA cohort clinical data were accessed using the cBioPortal [16, 17]. We used USCS XENA to acquire the normalized expression of PCMT1. Normalized gene expression was calculated as log2 (fpkm + 1). Cut-offs were calculated using the Cutoff Finder online tool [18]. The cutoff was set at 27.25 FPKM for the TCGA BRCA cohort and at 153 FPKM for TGCA TNBC cohort. Tumors with PCMT1 expression below the cut-off value were considered low-PCMT1; otherwise, they were classified as high-PCMT1.

### Statistical analysis

Statistical analysis was carried out using the Statistica 13.3 package (TIBCO Software Inc, California, USA). Based on the patient’s clinical data and the results obtained from the image analysis, the log-rank test was performed and the Kaplan–Meier curve was showed. Intergroup differences were calculated using the Mann-Whitney U test and ANOVA Kruskal-Wallis test. To determine the correlations between clinical variables and PCTM1 expression, Spearman’s rank correlation coefficient was used. We carried out univariate and multivariate analysis of potential predictors for OS using Cox proportional hazard regression. Results were presented as hazard ratio (HR). 95% confidence intervals (CI) were applied. To assess intergroup correlations, Chi^2 and t students were used. *p* value < 0.05 was considered statistically significant.

## Results

### Patient characteristics

The mean age of patients was 58.5 years (range 32–85 years). Most patients had low clinical stage disease, including 14 cases in stage IA, 39 in IIA, 8 in IIB, and 3 in IIIB. 15 (23.44%) tumors were classified as T1, 45 (70.30%) as T2, 2 (3.13%) as T3, and 2 (3.13%) as T4. The samples were categorized as low-stage (T1) and high-stage (T2–T4). 18 patients had lymph-node metastases and 9 patients had distant metastases at the time of diagnosis. The mean overall survival time was 60 months.

### PCMT1 overexpressed in breast cancer

**Fig. 2:**
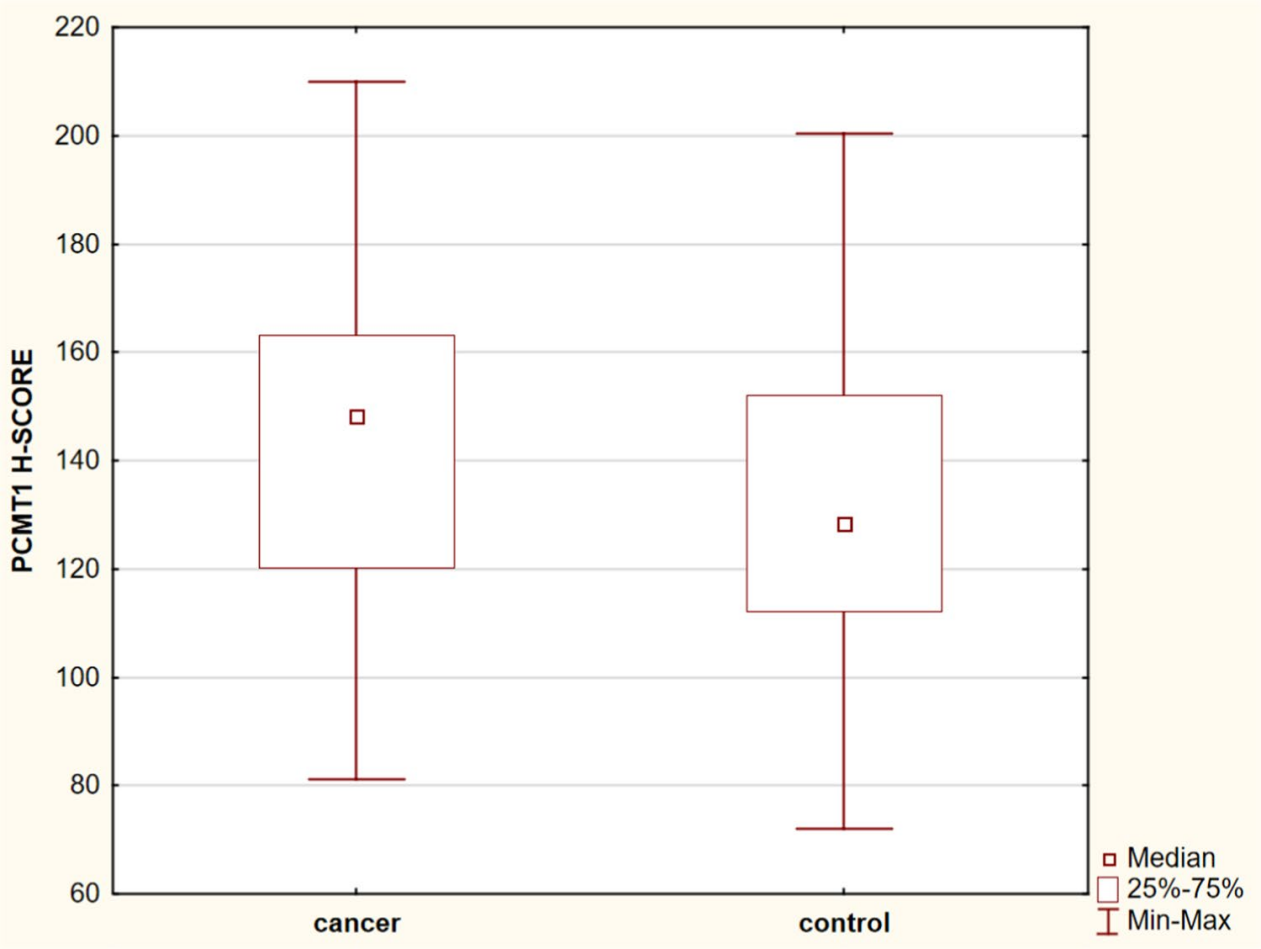
PCMT1 expression in cancer and control group (*p* = 0.036)

To find out the characteristics of PCMT1 staining patterns in breast cancer and the control group, we performed immunohistochemical staining using anti-PCMT1 monoclonal antibodies. PCMT1 expression was observed in all examined samples in both study and control groups. We observed strong PCMT1 immunoreactivity in breast-cancer samples, which was significantly higher than in the control group (median H-SCORE = 148 vs. 128.5, respectively, *p* = 0.036) (Fig. 2). PCMT1 was overexpressed in TNBC.

### PCMT1 expression correlates with cancer stage

PCMT1 expression was higher in T1 tumors when compared with T2-T4 tumors (*p* = 0.042). There were no significant differences in PCMT1 expression between groups of different lymph-node invasion status (*p* = 0.854), recurrence status (*p* = 0.927), or clinical stage (*p* = 0.627) (Table 2). We found no correlation between the type or received neoadjuvant or adjuvant therapy and the expression of PCMT1 (*p* = 0.33 and *p* = 0.44, respectively).

**Table 2:** Correlations between PCMT1 and clinical characteristics of study group

Variables	Total N	Median PCMT1 expression (Min–Max)	PCMT1 expression correlation	Statistical differences between groups
Cancer group	64	147.8 (81.2 -210.1)	0.744	*p* < 0.001
Control group	64	128.6 (72.1–200.3)		
Stage IA	14	138.2 (83.6–202.4)	0.164	*p* = 0.627
Stage IIA	39	148.5 (81.2–204.2)		
Stage IIB	8	151.9 (114.1–207.5)		
Stage IIIB	3	167.9 (82.1–210.1)		
T1	15	156.1 (112.8–207.5)	-0.250	*p* = 0.042
T2-T4	49	138.1 (81.2–210.1)		
N0	44	147.8 (81.2–210.1)	0.024	*p* = 0.854
N1-N3	18	147.9 (93.2–207.5)		
M0	57	148.2 (81.2–210.1)	-0.0529	*p* = 0.678
M1	7	142.9 (97.2–207.5)		
Recurrence	10	144.7 (93.2–172.3)	0.012	*p* = 0.927
No recurrence	54	148.3 (81.2–210.1)		

### Prognostic value of PCMT1 expression

To determine the prognostic value of PCMT1 expression in patients with TNBC, we divided the samples into low (41 patients) and high (23 patients) PCMT1 expression groups. The cutoff point was set at 153 and calculated using the Cutoff Finder online tool [18]. Univariate Cox regression analysis showed that high PCMT1 expression and cancer recurrence were significant prognostic factors of shorter survival. In multivariate analysis cancer recurrence remained statistically significant (*p* = 0.0002) (Table 3).

**Table 3:** Univariate and multivariate analysis of overall survival for study group

Variable	Univariate analysis			Multivariate analysis		
	RR	95% CI	*p* value	RR	95% CI	*p* Value
Stage (T1 vs. T2–T4)	0.79	0.22–2.85	0.724	–	–	–
Clinical stage (IA vs IIA–IIIB)	0.24	0.03–1.84	0.16	–	–	–
PCMT1 (low vs. high)	**0.26**	**0.09–0.79**	**0.0167**	0.199	0.02–1.50	0.1166
Lymph–node metastasis (N0 vs. N1–N3)	0.36	0.12–1.02	0.0545	–	–	–
Recurrence (Y/N)	**0.13**	**0.05–0.38**	**0.0002**	**0.133**	**0.04–0.39**	**0.0002**
Distant metastasis (M0 vs. M1)	0.76	0.17–3.42	0.726	–	–	–

Statistically significant results are bolded

**Fig. 3:**
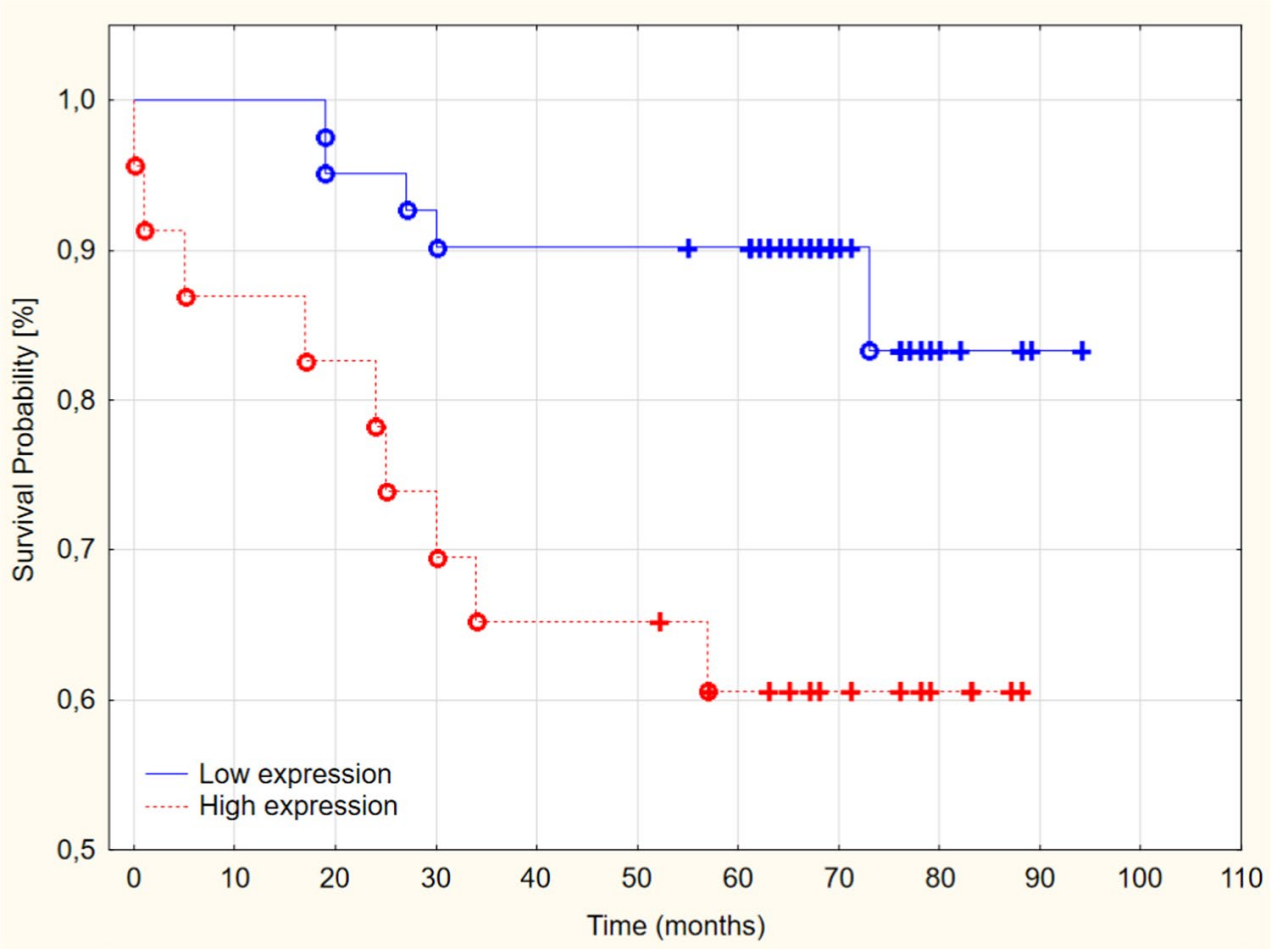
Overall survival in low and high-grade PCMT1 expression in a study group (*p* = 0.01)

The Kaplan–Meyer analysis presented that patients with high PCMT1 had significantly shorter survival and lower OS rate 5 years after being diagnosed than patients with low expression of PCMT1 (60.62% vs. 90.35; *p* = 0.01) (Fig. 3)(Table 4).

### TCGA breast cancer cohort

PCMT1 did not retain statistical significance as a prognostic factor in multivariate hazard analysis in our group, but due to *p* < 0.05 in univariate analysis and small size cohort size, which included only 4 (6.25%) patients with T3-T4 tumors, we decided to expand this study by including the analysis of a larger group (Table 5). We accessed The Cancer Genome Atlas (TCGA) database and obtained transcriptomics information through the Human Pathology Atlas [15]. The TCGA cohort consisted of 1062 cases with breast cancer (Table 6).

**Table 4:** Overall survival in low and high PCMT1 expression groups 1 year, 2 years and 5 years after diagnosis

	1-year OS [%]	2-years OS [%]	5-years OS [%]
PCMT1 Low	99.83	94.95	90.35
PCMT1 High	86.76	78.26	60.62

**Table 5:** Clinical characteristic of TCGA breast cancer cohort

Variables		*n* (%)
Age	Mean	58, 43 years (range 26–90)
Sex	Female	1062 (100%)
Stage	T1	178 (16.76%)
	T2	602 (56.69%)
	T3	240(22.59%)
	T4	19 (1.79%)
	Other	24 (2.26%)
Lymph-node metastases	N0	500 (47.08%)
	N1	349(32.86%)
	N2	119 (11.21%)
	N3	74 (6.97%)
	NX	21 (1.98%)
Distant metastases	M0	876 (82.49%)
	M1	21 (1.98%)
	MX	166 (15.63%)
Recurrence	Yes	100 (9.42%)
	No	777 (73.16%)
	Unavailable	185 (17.42%)
Disease course	Alive	910 (85.69%)
	Death	152 (14.31%)
Median follow-up time	28.27 months

**Table 6:** Univariate and multivariate analysis of overall survival in the TCGA breast cancer cohort

Variable	Univariate analysis			Multivariate analysis		
	RR	95% CI	*p* value	RR	95% CI	*p* value
Age (< 60 vs. > 60)	1.03	1.02–1.04	0.000001	1.04	1.02–1.06	0.00003
Stage (T1 vs. T2–T4)	0.45	0.27–0.76	0.003	0.58	0.25–1.38	0.22
PCMT1 (low vs. high)	1.02	1.01–1.03	0.00003	1.02	1.00–1.04	0.0499
Lymph node metastasis (N0 vs. N1–N3)	0.45	0.31–0.64	0.00001	0.88	0.51–1.51	0.643
Recurrence (N/Y)	0.13	0.09–0.20	< 0.000001	0.12	0.07–0.20	< 0.000001
Distant metastasis (M0 vs. M1)	0.2	0.12–0.34	< 0.000001	0.75	0.31–1.84	0.53

**Fig. 4:**
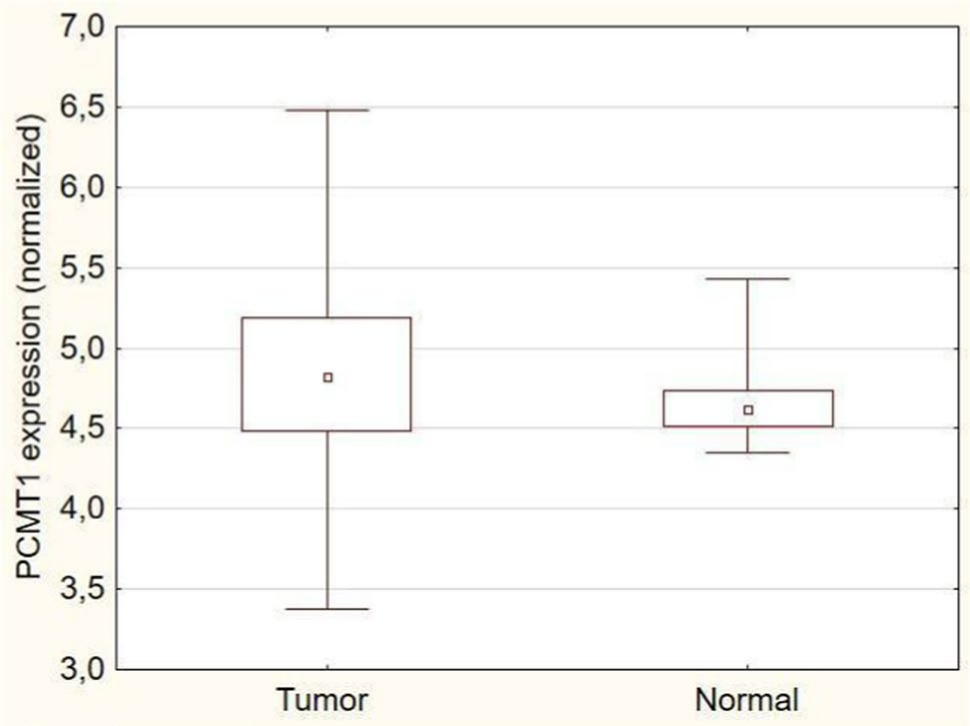
PCMT1 expression in cancer and control group in TCGA breast cancer cohort

PCMT1 expression in breast cancer samples was significantly higher in the cancer group than in the control group (median H-SCORE = 4.824 vs. 4.618, respectively, *p* = 0.0004) (Fig. 4).

The samples were dichotomized into low expression and high expression groups based on the FPKM (number of fragments per kilobase of exon per Million reads) value of PCMT1.) Univariate Cox regression analysis showed that expression of PCMT1, age, stage, lymph-node metastasis, distant metastasis, and recurrence were statistically significant prognostic factors of patients overall survival. Moreover, multivariate Cox regression analysis indicated that age, high PCMT1 expression, and recurrence were statistically significant prognostic factors of overall survival in the TCGA breast-cancer cohort (Table 6).

In luminal A and luminal B breast cancers, PCMT1 levels were higher in samples from patients older than 55 years of age compared to younger patients. We found no correlation between PCMT1 expression and clinical characteristics, such as tumor stage or lymph-node status, in any of the subtypes of breast cancer studied.

**Fig. 5:**
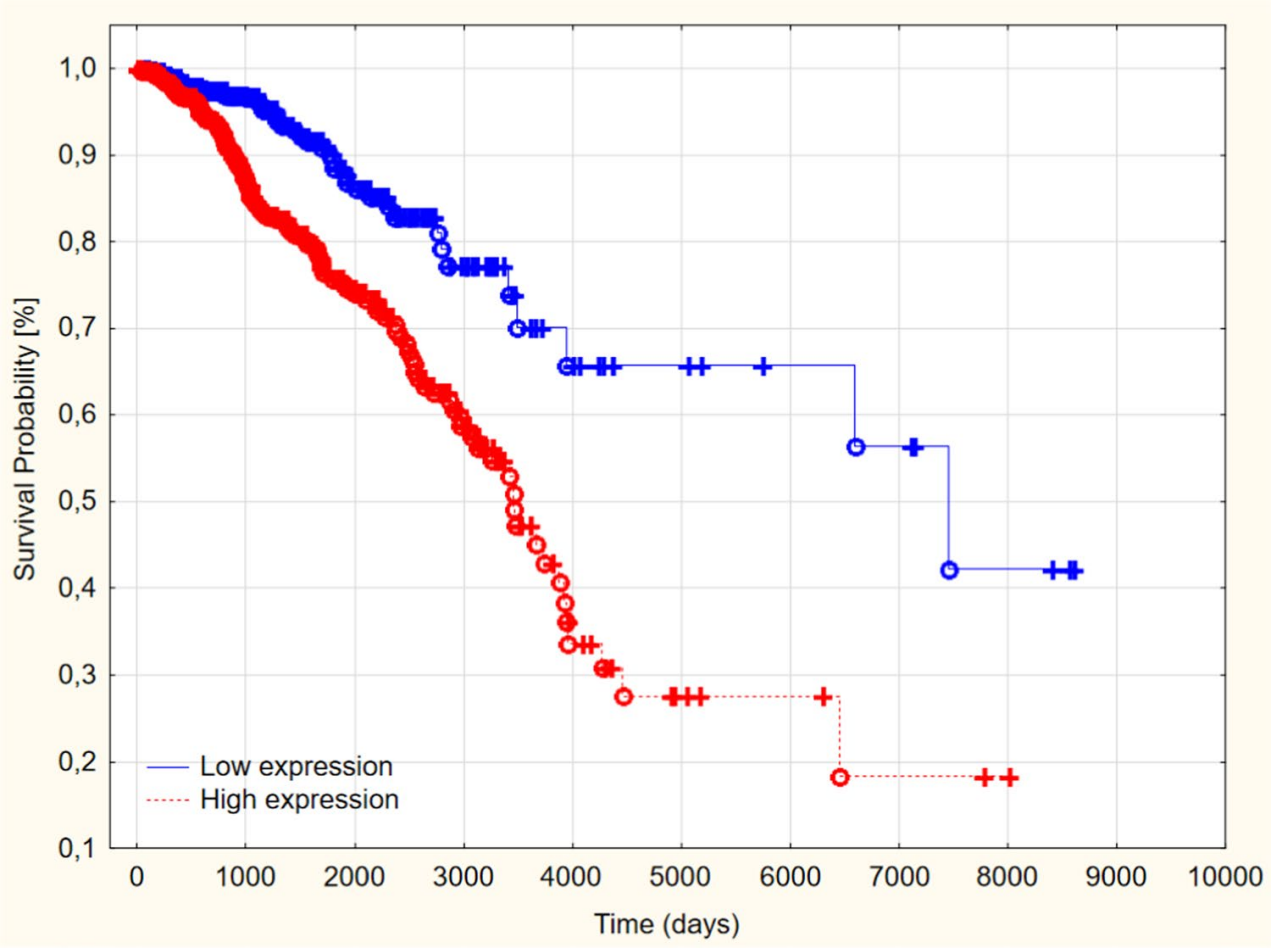
Overall survival in low and high-grade PCMT1 expression in TCGA groups (*p* < 0.005)

To determine the prognostic value of PCMT1 expression in patients with breast cancer, we divided the samples into low and high PCMT1 expression groups, with the cutoff point as 27.25. The 5-year survival rate in patients with high PCMT1 expression reached 76% and was significantly lower than the 89% 5-year survival rate in the low PCMT1 expression group (*p* < 0.005) (Fig. 5).

PCMT1 expression was prognostic of OS in the TCGA cohort, and its high expression correlated with shorter OS in breast cancer (Table 7).

**Table 7:** Correlations between PCMT1 expression and clinical characteristics in TCGA breast cancer cohort

BRCA subtype	Variables	Total N	Median PCMT1 expression (Min–Max)	Intergroup differences
TNBC	Age < = 55	93	30.8 (9.9–74.5)	*p* = 0.175688
	Age > 55	76	27.5 (4.7–69.5)	
	Race White	106	29.3 (4.7–74.5)	*p* = 0.6557
	Race Black/African American	52	27.6 (15.4–64.2)	
	Race Asian	6	33.4 (19.3–64.5)	
	T1	22	25.7 (9.9–40.6)	*p* = 0.0846
	T2	120	29.05 (4.7–74.5)	
	T3	21	31.7 (15.4–59.1)	
	T4	2	25.1 (17.4–32.8)	
	N0	106	27.15 (4.7–69.5)	*p* = 0.3390
	N1	46	30.35 (14.8–74.5)	
	N2	12	27.6 (15.4–59.1)	
	N3	5	32.9 (25.9–46.9)	
Luminal A	Age < = 55	189	24.7 (6.6–60.3)	*p* = 0.000002
	Age > 55	295	28.9 (9.4–72.1)	
	Race White	381	27.8 (6.6–72.1)	*p* = 0.7290
	Race Black/African American	54	26.4 (10.4–47.9)	
	Race Asian	20	29.8 (14.1–45.2)	
	T1	108	28.65 (6.6–72.1)	*p* = 0.7787
	T2	262	27.1 (9.4–67.5)	
	T3	102	28.55 (14.1–67.6)	
	T4	7	27.30 (24.6–35.7)	
	N0	233	27.8 (6.6–72.1)	*p* = 0.4762
	N1	165	26.5 (11.9–65.2)	
	N2	51	28.7 (16.7–67.6)	
	N3	34	28.85 (15.0–45.8)	
Luminal B	Age < = 55	77	31.5 (13.8–85.3)	*p* = 0.011353
	Age > 55	112	37.55 (10.7–95.1)	
	Race White	126	33.15 (10.7–95.1)	*p* = 0.8144
	Race Black/African American	26	34.25 (14.9–67.3)	
	Race Asian	16	35.6 (17.6–48.6)	
	T1	23	26.5 (13.8–59.8)	*p* = 0.1699
	T2	105	33.4 (14.9–95.1)	
	T3	57	36.8 (10.7–78.8)	
	T4	4	32.85 (28.0–54.5)	
	N0	75	33.5 (13.8–95.1)	*p* = 0.1660
	N1	68	32.55 (14.9–84.1)	
	N2	34	38.35 (10.7–78.8)	
	N3	10	39.7 (24.1–51.1)	
HER2 +	Age < = 55	32	29.4 (12.2–100.4)	*p* = 0.232188
	Age > 55	42	33.45 (14.5–66.5)	
	Race White	36	34.25 (12.3–100.4)	*p* = 0.2039
	Race Black/African American	15	25.3 (12.2–49.3)	
	Race Asian	14	34.25 (15.8–49.8)	
	T1	7	35.1 (19.8–52.1)	*p* = 0.7579
	T2	45	30.9 (12.2–100.4)	
	T3	18	30.75 (15.8–66.5)	
	T4	3	43.0 (26.8–48.7)	
	N0	26	30.15 (12.2–52.1)	*p* = 0.2429
	N1	27	33.8 (17.7–100.4)	
	N2	11	26.0 (15.8–66.4)	
	N3	7	27.4 (21.4–43.0)	

### TCGA TNBC cohort analysis

Next, we analyzed the triple-negative breast cancer cases extracted from the TCGA database. We found no correlations between PCMT1 levels, patients age, sex, race, tumor stage, lymph-node involvement status or distant metastasis (*p* > 0.05). In univariate analysis, tumor stage, lymph-node status, and PCMT1 levels were predictive of patients survival (Table 8). Multivariate Cox regression analysis confirmed that stage and PCMT1 expression were independent prognostic factors of overall survival in the TCGA TNBC cohort.

**Table 8:** Univariate and multivariate analysis of overall survival for TCGA triple-negative breast cancer cohort

Variable	Multivariate analysis			Multivariate analysis		
	RR	95% CI	p value	RR	95% CI	p value
Age (≥ 55 years vs. > 55 years)	0.7	0.3–1.67	0.42	-	-	-
Stage (T1 vs. T3-T4)	**0.17**	**007–0.42**	**0.001**	**0.25**	**0.08-.074**	**0.01**
Lymph-node status (N0 vs. N1–N3)	**0.24**	**0.09–0.63**	**0.003**	0.55	0.18–1.74	0.31
PCMT1 (low vs. high)	**0.19**	**0.06–0.64**	**0.007**	**0.23**	**0.07–0.81**	**0.02**

**Fig. 6:**
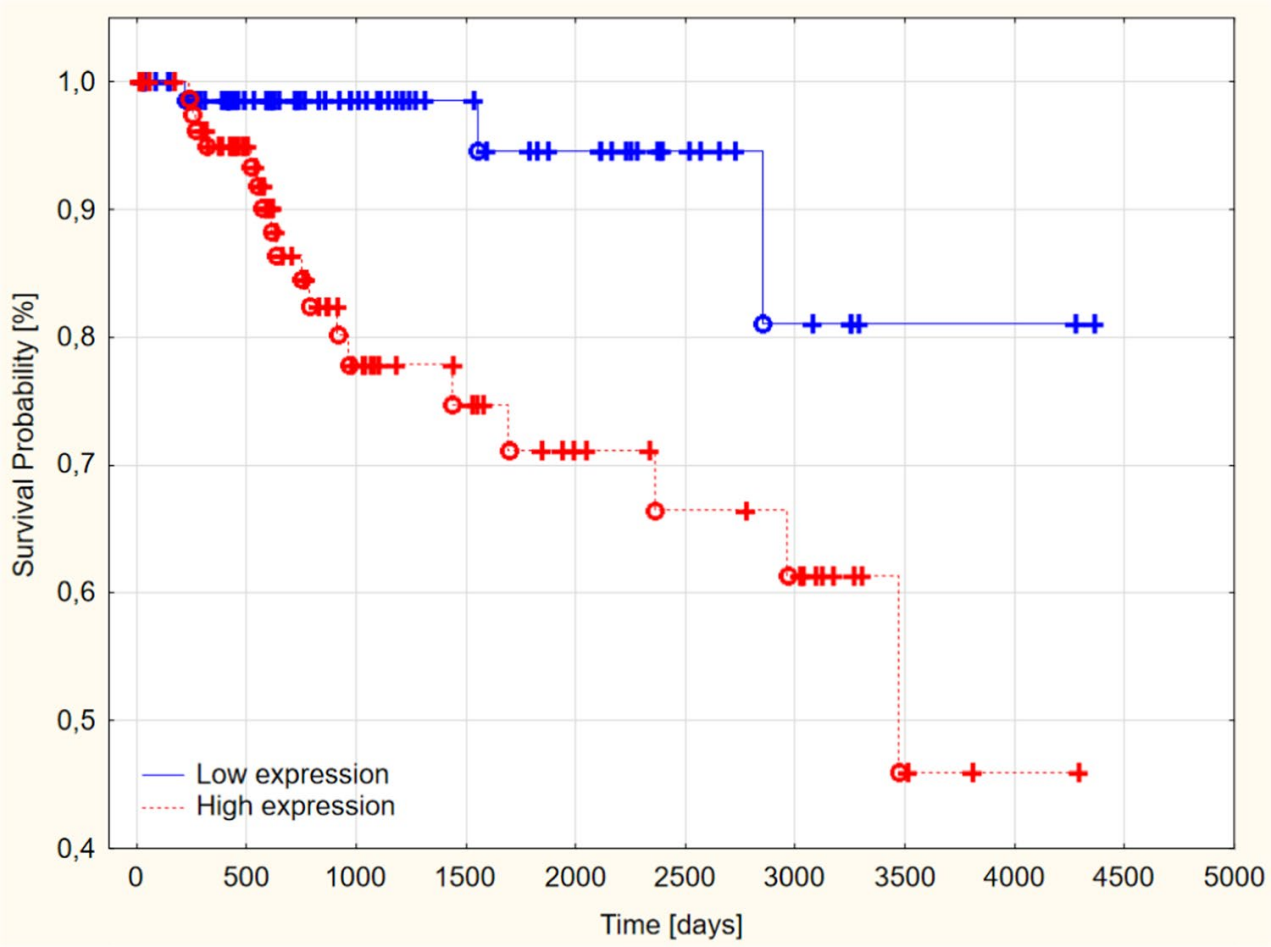
Overall survival in low and high-grade PCMT1 expression in TCGA TNBC groups (*p* < 0.005)

The 5-year survival rate in patients with high PCMT1 expression reached 71% and was significantly lower than the 95% 5-year survival rate in the low PCMT1 expression group (*p* < 0.005) (Fig. 6). PCMT1 expression was a prognostic in the TCGA TNBC cohort, high expression predicts shorter overall survival in triple-negative breast cancer.

## Discussion

Due to its aggressive course and poor prognosis TNBC remains of high interest to many scientists. Our study showed that the overexpression of the PCMT1 protein is associated with a worse prognosis and reduced overall survival in patients with TNBC, suggesting that PCMT1 may be a prognostic marker in this disease [12]. Similar conclusions were reached by other authors, who concluded that PCMT1 overexpression in bladder cancer was an unfavorable prognostic factor for patient survival [4]. PCMT1 overexpression was also associated with shorter survival in breast cancer [12]. In addition, overexpression of the PCMT1 was correlated with worse prognosis in cervical cancer [10].

To compare our results with other types of breast cancer, we used the Human Protein Atlas database and evaluated the overexpression of PCMT1 in clinical breast-cancer samples. The results showed a similar correlation—PCMT1 was overexpressed in breast cancer cells. This suggests that PCMT1 may function as a prognostic biomarker for survival in selected patients, who could be offered more intensive treatment and more frequent monitoring [15].

In contrast to our study, PCMT1 protein overexpression is positively correlated with metastasis among ovarian and bladder cancer patients, indicating the biologic function of PCMT1 in regulating cell proliferation, migration and invasion in cancer cell lines [4, 7, 13]. The lack of correlation between metastases and PCMT1 expression in our study may be related to post-translational modifications and different protein expression processes present in the tested TNBC samples [9]. Furthermore due to to a small number of subjects with disseminated and metastatic TNBC in our group and the TCGA database, further research are necessary.

**Fig. 7:**
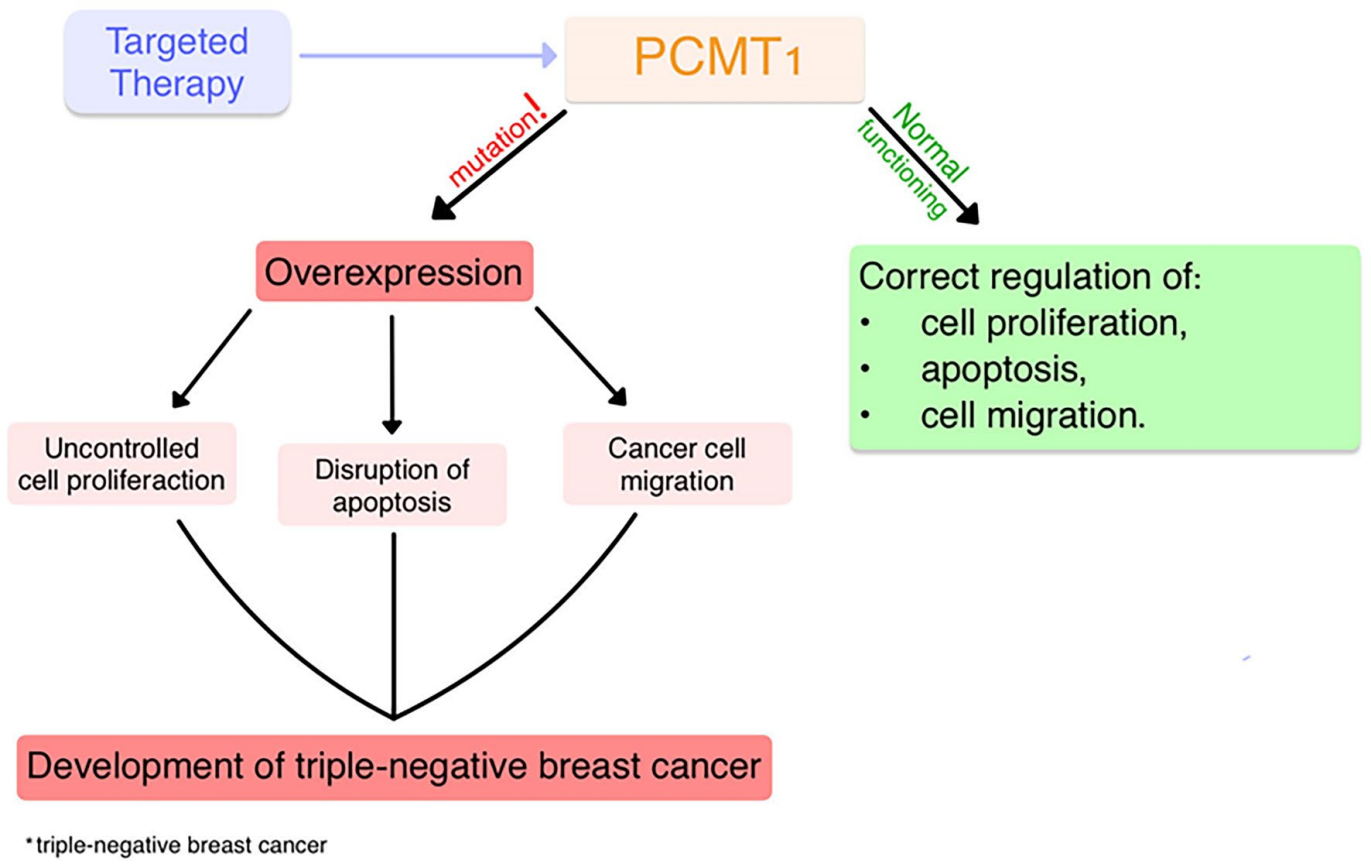
Suggested role of PCMT1 in TNBC development

Recently, Guo et al. reported a positive correlation between PCMT1 overexpression and tumor stage in breast cancer [12]. We also found that PCMT1 levels increased along TNBC tumor stage. However, Dong et al. did not found a similar correlation in their bladder cancer study [4]. Additional studies show that the inhibition of PCMT1 induced apoptosis and suppressed cell proliferation and invasion in breast-cancer cells. Those reports indicate that targeting the regulatory SNHG16/miR-195/PCMT1 axis could serve as a promising therapeutic target for patients with breast cancer.9 PCMT1 regulated the proliferation, apoptosis, and migration of various cells (Fig. 6). Mutations that cause its overexpression disrupt the fragile balance between anti-apoptotic and pro-apoptotic proteins, driving uncontrolled proliferation and contributing to disease progression. PCMT1 overexpression in cancer cells can impairs the repair of proteins in the tumor microenvironment and hinder immune anticancer response. Therefore, the use of targeted therapy that inhibits PCMT1 pathways may suppress the development of TNBC. For instance, PCMT1 knockdown in liver cancer cells could promote their apoptosis while also limiting their proliferation and survival [19]. Furthermore, since PCMT1 overexpression could reduce the infiltration of tumor-killing immune cells, therapies aimed at reducing PCMT1 levels may oppose cancer immunoediting and enhance host antitumor response (Fig. 7). However, further research are necessary to ensure a smooth switch from preclinical to clinical settings [9, 20].

## Limitations

The limitation of this study was the unproportional amount of early-stage cancer patients, which had an impact on conducting statistical analysis. The TNBC study group consists of only 64 patients, therefore we decided to expand our analysis by the TCGA Breast Cancer Cohort.

## Conclusions

PCMT1 is overexpressed in TNBC which is associated with significantly shorter overall survival of patients in both the study group and TCGA Breast Cancer Cohort. PCMT1 expression is positively correlated with TNBC cancer stage, but there is no significant correlation between PCMT1 expression and lymph-node metastasis, progression and recurrence status. PCMT1 overexpression may lead to uncontrolled cell proliferation, disruption of apoptosis and cancer cell migration during TNBC development. As a result of all this, PCMT1 may be a potential prognostic biomarker for patients with TNBC, but further research is needed to find out clinical applicability of PCMT1 in TNBC treatment.

## Data Availability

TCGA cohort clinical data are openly available in cBioPortal [16, 17]. Study group clinical data may be shared by corresponding author on reasonable reason.
